# Risk Predictability in Early Life Shapes Personality of Mosquitofish in Adulthood

**DOI:** 10.3390/ani13071214

**Published:** 2023-03-30

**Authors:** Mengdi Si, Wenwen Zhang, Chunlin Li, Li Su, Xinyu Zhang

**Affiliations:** 1 School of Resources and Environmental Engineering, Anhui University, Hefei 230601, China; 2 Anhui Province Key Laboratory of Wetland Ecosystem Protection and Restoration, Anhui University, Hefei 230601, China; 3 Anhui Shengjin Lake Wetland Ecology National Long-Term Scientific Research Base, Dongzhi 247230, China; 4 Ministry of Ecology and Environment, Nanjing Institute of Environmental Sciences, Nanjing 210042, China

**Keywords:** animal personality, behavioral syndromes, repeatability, risk predictability

## Abstract

**Simple Summary:**

Animal personality refers to consistent behavioral differences among individuals, which have been found to exist in many animal species. Although personality is largely determined by genetic factors, it might be greatly influenced by external environmental factors, e.g., risk pattern, which is still poorly known. In this study, newborn mosquitofish, *Gambusia affinis*, were reared to sexual maturity under different treatments of risk predictability (i.e., no risks, unpredictable risks, risks at 5 min after feeding and risks at 2 h after feeding) to investigate the effect of risk predictability in early life on their personality traits (i.e., shyness and exploration). We found that mosquitofish’s exploration showed repeatability in all risk treatments, while the shyness of the fish was repeatable only in the predictable risk treatments. Shyness was negatively related to exploration in the three risky treatments, no matter predictable or unpredictable. The fish reared under predictable risk were less explorative, and we did not find differences in shyness across treatments. These findings suggest that risk predictability in early life may be important in shaping animal personality.

**Abstract:**

Animal personality is of great ecological and evolutionary significance and has been documented in many animal taxa. Despite genetic background, personality might be prominently shaped by external environments, and it is significant to explore the environmental factors that influence the ontogeny of animal personality in early life. Here, we reared newborn mosquitofish *Gambusia affinis* under different treatments of risk predictability (i.e., no risks, unpredictable risks, risks at 5 min after feeding and risks at 2 h after feeding) and measured their two personality traits at sexual maturity. We measured the behavioral repeatability, correlation between behavioral characteristics, and the impact of risk predictability. We found that the fish showed repeatability in exploration in all risk treatments, as well as repeatability in shyness under predictable risks. When growing up in risk treatments, no matter predictable or unpredictable, shyness and exploration showed a negative correlation, suggesting a behavioral syndrome between the two behavioral traits. The fish reared under predictable risks were less explorative than those under unpredictable risks, while there were no differences in shyness among treatments. Besides, smaller fish were bolder and more explorative than larger ones. Our findings imply that risk predictability in early life may play an important role in shaping animal personality and modifying the average behavioral levels.

## 1. Introduction

Animals have been found to behave consistently within individuals and differently among individuals across different contexts and over time, which is defined as animal personality [1,2]. It is a significant source of intraspecific variation, and it has been found to widely exist in invertebrates and vertebrates, thus attracting increasing theoretical and empirical research attention [1,3]. Some important ecological and evolutionary processes might be related to animal personality, e.g., foraging behavior [4], mate choice [5], intraspecific interactions [6], spatial dynamics of populations [7], and speciation [8].

Although research has shown that personality and its associated behavior and physiological characteristics are heritable [9], personality might be largely influenced by internal and external factors [3,10]. Researchers have demonstrated that individuals reared within the same social group may exhibit dissimilar personality traits as a result of variations in sex [11], body size [12], and energy metabolism status [13]. For example, smaller animals are generally found to be bolder and more active than their larger counterparts [14,15]. Animals living in various environments experience different external factors, such as environmental complexity [16], food availability [17], and predation risk [18], and these factors may also shape personality traits. Individuals may also develop distinct personality types when exposed to different microenvironments [19,20]. Among the various environmental factors, predation risk can occur throughout the lifetime of prey animals and is strongly related to their survivals [21]. Because of the frequent interactions between prey and predators, predation risks may have significant effects on prey’s personality, which can largely influence prey’s fitness.

Predation risk is an important selective environmental factor that can drive the evolution of animal behavior and morphological characteristics [22,23]. Prey animals are affected by predation risk in a variety of characteristics from physiology to behavior, which can lead to a range of ecological consequences [24,25,26]. When encountering predators, damselflies, *Enallagma vesperum,* increase levels of arginine kinase to raise their swimming speed and escape ability [27]. Previous studies mostly focused on the effects of risk presence [28] or risk levels on animal behavior [29]. However, risk is by nature highly variable over time, making the temporal variation potentially important for prey’s responses to predation risk [23]. For example, convict cichlids living in low-risk environment have longer movement time when the risk is predictable than unpredictable [30]. Animal personality is the composite result of heredity and long-term responses to environment [31], and the environment may vary during animals’ lifetime. Throughout the lifetime of prey animals, predation risk often occurs unpredictably [22]. Long-term behavioral responses to unpredictable risks may largely shape personality trait, which has been rarely studied.

Environmental influences on personality can last across the animals’ lifetime and may vary depending on developmental stages [3]. It is recommended to focus on the ontogenetic “sensitive window”, during which living conditions have strong influences on animal behaviors [32]. The experience during early sensitive stages can greatly mediate animal behavior, which would be confirmed during the consolidation process [33]. Adolescence is an important stage for the ontogeny of behaviors and adult behaviors are largely determined during this stage [34]. As a behavioral characteristic, animal personality has been found to be shaped by experiential factors during early life stages and remains highly consistent in adults, generating long-lasting effects on adult life [35]. Young animals are often susceptible to predation risks, and their responses can determine their fate and fitness [36]. Personality might be shaped during this process, forming certain behavioral strategies as responses to predation risks. Therefore, exploring the effects of predation risks on personality in early life may assist in comprehending development of animal personality and the associated adaptive consequences.

In this study, we investigated the influence of risk predictability during early life on personality of adult mosquitofish, *Gambusia affinis*. The native distribution range of mosquitofish is the North America, and it has become a famous invasive species all over the world [37]. It is highly adaptable and able to utilize a variety of natural and artificial environments, which makes it the most widespread freshwater fish [38]. Mosquitofish is a teleost, belonging to Poeciliidae, with the characteristics of ovoviviparity, internal fertilization, and sexual dimorphism [39]. At sexual maturity, the female fish have a dark gravid spot on each side of their abdomen, while the males have a gonad with barbs and spines at the tip modified from the anal fin [37].

We reared newborn mosquitofish under temporally predictable and unpredictable risks until sexual maturity and measured their two personality traits, i.e., shyness and exploration. The repeatability of the two behavioral traits and the correlation between them were tested separately in different treatments. We hypothesized that fish reared under predictable risks would exhibit behavioral repeatability because their regular responses to the temporal pattern of risks may result in behavioral rhythm [40]. Behavioral correlations may be adaptive in risky environments [41,42], so we predicted behavioral syndromes in the risk treatments, no matter whether they are predictable or unpredictable. When living under predictable risks, animals might be more certain that risks would occur [43]. Therefore, we expected fish growing under predictable risks to be shyer and less explorative than those under unpredictable risks.

## 2. Materials and Methods

### 2.1. Study Animals and Rearing

The subjects of this study were the offspring of a wild mosquitofish population collected in a farmed pond in Shanghai (120.99° E, 31.01° N) in June 2021. We captured 300 gravid females using nets and brought them to the laboratory at Anhui University, where they were evenly kept in 20 tanks (37 cm long × 27 cm wide × 13.5 cm high) containing aerated tap water at about 26 °C. Fine-grain commercial food (TIDDLER, Weifang YEE Pet Products Co., Ltd., Weifang, China; crude protein 42%, crude fat 5%, crude fibers 5%, ash 11%) were provided to the mosquitofish at 8 A.M. and the fish were placed in a natural photoperiod (~14: 10 L: D).

We regularly checked the mother’s tanks and collected 800 newborns in a week. The newborns were evenly reared in 32 net tanks (blocks: 26 cm long × 15 cm wide × 15 cm high; mesh size: 0.5 mm) in oxygenated tap water with a depth of 8 cm. To avoid potential genetic effects, we randomly and equally divided the fry into the blocks. Due to this allocation method, the sex ratio of fry and the number of mothers contributing to the fry were assumed to be roughly equal in each block. In the rearing process, the water temperature was between 25~30 °C, and the pH was 7.54~7.76. The fish were exposed to a 14:10 light:dark photoperiod, and the feeding time was from 8 to 9 A.M. each day. They were offered brine shrimp nauplii and rotifers within 14 days of birth, then they were offered fine-grained commercial food.

The 32 net tanks were divided into four groups under different treatments of risk predictability: no risks, unpredictable risks, risks at 5 min after feeding, and risks at 2 h were considered after feeding. In the two predictable treatments, risks were initiated at 5 min and 2 h after feeding, respectively. We divided the time from 8 a.m. to 10 p.m. into 168 five-minute slots that were continuously numbered. A random serial number was chosen to exert the risks for the unpredictable group in each day. To avoid the potential habitual effects of the same risk, we simulated five kinds of risks: hitting the net tanks with a stick, throwing a leaf into the tanks, chasing the fish with a net, and throwing a plastic ball or foam board, which were found to induce fish’s avoidance behavior in preliminary experiments. The five risks were used one by one, and each certain risk was performed for approximately 2–3 s (according to the preliminary experiment) once in a day, and the frequency of risk was the same among the risk treatments.

In September, when the mosquitofish reached sexual maturity, we randomly captured the subjects (body length >15 mm) for the following behavioral experiments. Due to the small number of males, we only tested female fish. A total of 128 female individuals, four in each block, were randomly selected, and they were kept separately in opaque black labeled containers (height: 9 cm; diameter: 15 cm; hereafter, the holding container) equipped with aerated tap water. We put an opaque cylindrical black refuge chamber (height: 5 cm; diameter: 7 cm) in the middle point of the container. They were allowed a minimum of one day to adapt to the container before experiments. The fish were under a natural photoperiod and were not allowed to receive food 12 h before the behavioral tests.

### 2.2. Personality Assays

**Figure 1:**
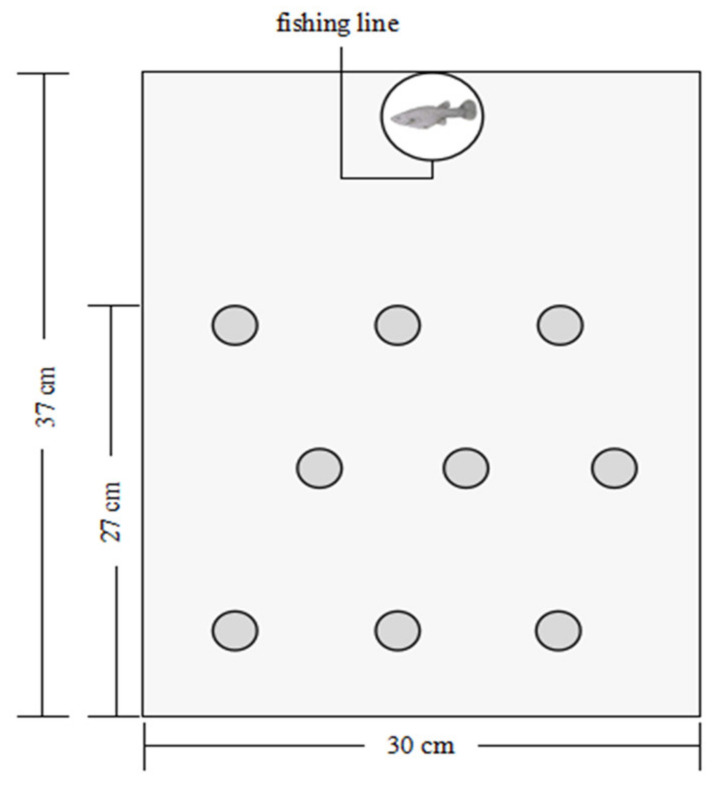
Schematic diagram of the experimental system.

A plastic white non-transparent tank (37 cm long × 30 cm wide × 20 cm high) was used as an arena to measure shyness and exploration in a laboratory with sufficient light and constant temperature (26 °C) during the light of 14:10 light:dark photoperiod (Figure 1). Background colour may cause stress to fish, but with mixed effects [44]. We tried using arenas of other colours, but we found no significant differences in their influences on the fish behaviors. Compared with other colours, white has an advantage in helping observers to easily and accurately locate the fish in the arena.

The arena contained 3 cm of oxygenated tap water, which was replaced after each subject was tested. One end of the arena was fixed with a cylindrical black opaque chamber (height: 5 cm; diameter: 7 cm) to be used as a shelter for the subject (hereafter, starting refuge). Novel objects were placed in fixed locations of the remaining space of the arena to prevent unobstructed viewing of the environment by the fish. Different materials, i.e., plastic flakes, plastic artificial leaves, and gravel, were used as new objects for different trials on the same fish to prevent possible habituation effects on the identical objects. The starting refuge was equipped with a sliding trapdoor (3 cm long × 3 cm wide) that could be remotely opened via a fishing line to allow fish to enter the arena. During the experiment, there was a camera (Sony HDR-CX510, 55× extended zoom, Sony Corporation, Tokyo, Japan) over the stage to capture the subject’s movement. An opaque curtain was used to shield the experimenter to avoid any disturbance to the tested fish during experiments. Each fish was tested three times over three consecutive days, after which we measured its body length (accurate to 0.1 mm).

At the start of the experiment, a fish was randomly selected and carefully placed in the shut starting shelter, and the camera was activated. The subject had a 5-min acclimation period, after which the trapdoor was opened remotely. The shyness of the subject was measured as the time it took to emerge from the starting refuge (i.e., latency). Latency represents the willingness of the fish to leave the refuge to explore unknown environments [45]. When a fish’s whole body passed the trapdoor, we considered that it had come out of the starting refuge. After the subject left the refuge, the camera kept documenting for 10 min (movement video). Then, the tested fish was returned to its holding container. A total of 600 images (one frame per second) were intercepted in the 10-min movement videos. Image J (http://rsbweb.nih.gov/ij/; accessed on 2 November 2021) was used to track the position of the head of the mosquitofish in the image, using the x/y coordinates of the head per second to depict its movement pathway. Consistent with Li et al. [46] and Xu et al. [16] and analogous to Edenbrow and Croft [47], total path length was used as the exploration score of the fish. Since animals should be active when exploring, we acknowledged that the assessment of exploration may include components of activities [48]. However, given that the test environment was unfamiliar for the fish, we referred to it as an exploratory behavior, that is, the distance covered in the arena [1,49].

### 2.3. Ethical

The experiments followed the China’s current animal welfare and research ethics regulations. All animal care and experimental programs were endorsed by Anhui University’s Institutional Animal Care and Use Committee (permission no. 2020-033). These fish suffered no harm in the experiments. After the experiment, we transferred the fish to a pond on the campus of Anhui University where mosquitofish were present for several years [16].

### 2.4. Statistical Analyses

Consistency of individual behavior is often gauged by repeatability [50]. The function *rpt* in the R package *rptR* [51] was employed to measure the repeatability of shyness and exploration in each treatment of risk predictability, and body length was used as a fixed effect, while individual ID was used as the random effect. To estimate confidence intervals, the number of parametric bootstrap iterations was controlled by setting the *nboot* argument to 1000 bootstraps. The *p*-values based on likelihood ratio tests were used to determine the significance of behavioral repeatability. The two behavioral traits were averaged, respectively, to be used in correlation test, and Shapiro-Wilk was used to test whether the data were normally distributed. The Spearman rank correlation was performed using the *corr.test* function in the package *psych* [52] to test the correlation between the two behaviors, after finding that the means were not normally distributed.

Each behavioral characteristic was fitted using a generalized linear model (GLM) that had a Gaussian error structure to examine the effects of risk predictability and body length on each behavioral trait. The initial model contained an interaction term, which was finally removed because the interaction effect was not significant determined by the confidence interval including. The *emmenas* package was used to make post hoc comparisons among different risk treatments. The statistical tests were carried out using R v.3.6.3 [53].

## 3. Results

### 3.1. Behavioral Repeatability and Correlation

**Figure 2:**
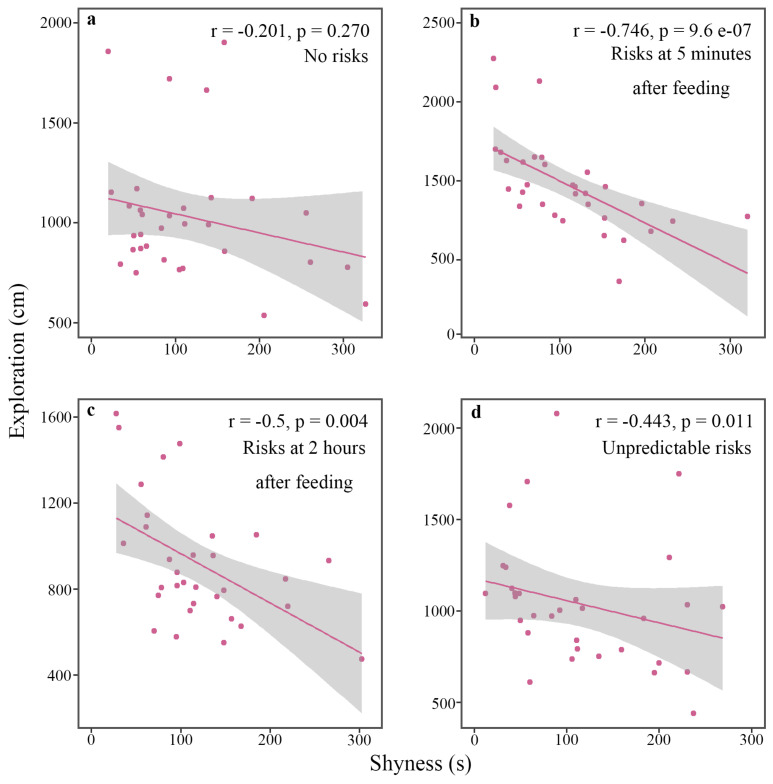
Correlation between shyness and exploration of female mosquitofish reared under no risks (**a**), risks at 5 min after feeding (**b**), risks at 2 h after feeding (**c**), and unpredictable risks (**d**).

The tested fish showed significant repeatability in exploration in all risk treatments, and repeatability in shyness in the two predictable treatments was observed (Table 1). There was a negative correlation between shyness and exploration in risk treatments, no matter predictable or unpredictable (Figure 2).

### 3.2. Effects of Factors on Behavioral Traits

All subject individuals left the refuge in a maximum of 11 min, with bolder individuals having shorter latency. There were no differences in shyness among the fish reared under different risk predictability. The fish reared under unpredictable risks showed higher levels of exploration than those reared under predictable risks. The average body length of the tested fish was 20.0 ± 2.1 mm (mean ± SD), and smaller fish were found to be bolder and more explorative (Table 2 and Table 3).

## 4. Discussion

Our study further indicates the presence of personality and behavioral syndromes of mosquitofish, and this indicates a role of risk predictability in early life in influencing their personality traits (Table 1 and Table 2, Figure 2). Animal personality refers to consistent behavioral differences between individuals of the same species [49,54]. Differences in behavior among individuals were found to explain about 37% of phenotypic behavioral differences within populations [50]. Animal personality may affect the survival and fitness of particular individuals, and thus this may have important ecological and evolutionary implications [10,14]. Studying the ontogeny of animal personality might contributes to understanding of the underlying mechanisms of variation in behavior among individuals and the adaptive significance of different behavioral strategies among individuals facing stressful challenges [1].

We found that mosquitofish showed repeatability in exploration under all risk treatments (Table 1). Repeatability may influence the behavioral tactics of animals (e.g., breeding) and thus their fate and life history [55]. Previous studies have found that exploration is repeatable in a large number of animal species [16,56,57]. Exploration is a basic behavior of animals, helping them to gain more comprehensive information about the environment [58]. The widespread presence of repeatability indicates that animals have limited plasticity in this behavior, benefiting them by reducing costs of changing behavioral strategies in various environments [56]. The fish showed repeatable shyness only when they were reared under predictable risks. Shyness is an individual’s response to risks [59], so the effect of predation risk on shyness is more direct than exploration. The fish reared under predictable risks experienced risk stimuli at a fixed time each day, which may help them develop stable behavioral responses to these stimuli, i.e., personality. In contrast, animals may underestimate the risk level in unpredictable environments [43], which may not help strengthen behavioral repeatability. The underestimated risk level may also explain why the fish did not exhibit repeatable shyness in treatments with no risks.

Shyness was negatively associated with exploration in all treatments with risks (Figure 2). Behavioral syndrome refers to a suite of related behaviors, and the behavior in one situation is related to the behavior in many other situations [28]. When behavioral traits are related, behaviors might not evolve independently [49]. Behavioral syndromes may exist because selection favors a range of related behaviors, acting on multiple behavioral phenotypes simultaneously [60]. Behavioral correlations can generate trade-offs (i.e., a trade-off between feeding and avoiding predators) that limit an animal’s ability to cope with its environment and therefore may affect individual fitness [28,49]. Behavioral syndrome may be shaped by various environmental factors, among which predation risk is a common factor for prey. Studies have found that predation risk as a selective pressure can reinforce the correlation between behaviors that might be influenced by predation risk. For example, compared with those in predator-free environments, animals living in predatory environments are found to have stronger correlation between aggression and exploration [42]. Our study also provides evidence for that the presence of risks may be conducive to the formation of behavioral syndromes.

As expected, mosquitofish grown under predictable risks were less explorative than those under unpredictable risks. Animals are predicted to reduce activities when the risk level is high. For example, the guppies, *Poecilia reticulata,* living in higher-risk environments are less explorative [61]. Animals may be more certain of the presence of risks when living in predictable risks, while those grown in uncertain environments may underestimate risk [62]. To avoid the perceived high risks, the fish reduced their explorations under predictable risks. Similarly, the lower perceived risk level in the unpredictable risks’ treatment may explain why the exploration under this environment did not differ from that under no risks. Predation risk may not only influence animals’ exploration, but also their shyness. Shyer individuals are risk averse and less susceptible to predation [1], so they may be more appropriate in high-risk environments. However, we found no difference in the shyness of mosquitofish among risk treatments. Previous studies have shown that the temporal predictability of risk has effects on individuals’ neophobic responses [63]. There were also some studies suggesting that the effect of temporal predictability may be influenced by other environmental factors [64], and females may not be highly sensitive to risks as males [65]. We only studied personality traits of females in this study, which may account for the nonsignificant difference among the treatments.

Consistent with some studies [66,67], we found smaller fish were more proactive, i.e., bolder and more explorative (Table 2). According to the metabolic hypothesis, smaller individuals have faster metabolic rates, so they need to emerge from shelters earlier and explore the environments more than larger ones [67,68]. As body size increases, reducing risk-taking behaviors in dangerous environments would be beneficial [12,67]. Therefore, the negative correlation between proactivity and body size might be a general pattern in prey. However, some other studies found the opposite pattern [60,69], and they explained that larger individuals might have stronger resistance ability when encountering predators [70,71]. There are also some studies reporting no effects of body size on animal personality [47]. These conflicting results suggest that body size and environmental factors may work together to influence the behavioral strategies of animals, and further studies are needed to investigate the underlying mechanisms.

Our results were generated from the analyses based on behavioral data of female mosquitofish, which might be different from those of males. However, we did not have enough male individuals (i.e., one to roughly two in each treatment) to obtain statistical significance. The sex ratio of the fish was found to be highly female-biased, even in natural populations (4.38:1; Öztürk and Ikiz [72]). The ratio bias might be more serious in experimental conditions, and the sex is difficult to be determined based on anal fins when the fish are young [73]. Although we reared a large number of newborn fish in each treatment (25 individuals), the average number of fish was nine in each rearing tank when they reached sexually maturity and the average proportion of males was small (5.06:1). Because of the potential behavioral differences among sexes, future studies should investigate how the effects of risk predictability on personality traits differ between males and females.

## 5. Conclusions

Our study further indicates that mosquitofish exhibit personality and behavioral syndromes. The repeatability of exploration was significant under all risk treatments, while shyness was only repeatable under predictability risks. Shyness and exploration were negatively correlated when the fish were grown under risks. Compared to those growing up under unpredictable risks, the fish under predictable risks were less explorative, which may be because that fish living under predictable environments could be more certain about the occurrence of risks. There was no significant difference in shyness among fish growing up in different levels of risk predictability, which might be attributed to the low risk intensity in this study. In addition, we found that smaller fish were bolder and more explorative, consistent with many other studies. Our results suggest that risk predictability during early life play an important role in influencing animal personality and modifying mean behavioral traits.

## Figures and Tables

**Table 1:** The behavioral repeatability of female mosquitofish reared under different risk predictability.

Risk	Repeatability	Shyness	Exploration
No risks	Repeatability values	0.174	0.478
	Standard error	0.111	0.104
	95% confidence interval	0, 0.391	0.26, 0.659
	*p*-value	0.078 ^•^	<0.001 ***
Risks at 5 min after feeding	Repeatability values	0.192	0.5
	Standard error	0.112	0.105
	95% confidence interval	0, 0.401	0.276, 0.678
	*p*-value	0.057 ^•^	<0.001 ***
Risks at 2 h after feeding	Repeatability values	0.253	0.539
	Standard error	0.112	0.100
	95% confidence interval	0.026, 0.469	0.301, 0.705
	*p*-value	0.012 *	<0.001 ***
Unpredictable risks	Repeatability values	0.18	0.475
	Standard error	0.11	0.11
	95% confidence interval	0, 0.401	0.244, 0.67
	*p*-value	0.071 ^•^	<0.001 ***

^•^*p* < 0.1, * *p* < 0.05, *** *p* < 0.001.

**Table 2:** The effects of risk predictability on the personality traits of female mosquitofish with body length as a covariate.

Personality	Variables	Estimate	SE	*t*-Score	*p*-Value
Shyness	Risks at 5 min after feeding	1.218	15.335	0.079	0.937
	Risks at 2 h after feeding	5.094	15.13	0.337	0.737
	Unpredictable risks	−8.171	15.306	−0.534	0.594
	Length	7.963	2.647	3.008	0.003 **
Exploration	Risks at 5 min after feeding	−85.738	55.870	−1.535	0.126
	Risks at 2 h after feeding	−115.407	55.124	−2.094	0.037 *
	Unpredictable risks	35.445	55.765	0.636	0.525
	Length	−30.678	9.645	−3.181	0.002 **

* *p* < 0.05, ** *p* < 0.01.

**Table 3:** Means of body length and behavioral traits (±standard deviation, SD) of female mosquitofish reared under different risk predictability.

Risk	Sample Size	Body Length (mm)	Shyness (s)	Exploration (cm)
No risks	32	20.1 (±1.8)	115.8 (±118.6)	1031.0 (±404.8)
Risks at 5 min after feeding	32	19.1 (±2.1)	109.4 (±101.0)	974.6 (±358.6)
Risks at 2 h after feeding	32	19.9 (±1.7)	119.7 (±91.3)	920.3 (±348.3)
Unpredictable risks	32	21.0 (±2.5)	114.7 (±110.7)	1039.3 (±428.1)

## Data Availability

The data presented in this study are available upon request from the corresponding author.
